# Talar component subsidence correlates with periprosthetic osteolysis after total ankle arthroplasty

**DOI:** 10.1007/s00590-023-03519-9

**Published:** 2023-03-17

**Authors:** Shinsuke Kihara, Noriyuki Kanzaki, Takahiro Yamashita, Tetsuya Yamamoto, Kyohei Nishida, Kanto Nagai, Daisuke Araki, Yuichi Hoshino, Takehiko Matsushita, Ryosuke Kuroda

**Affiliations:** 1 Department of Orthopaedic Surgery, Kobe University Graduate School of Medicine, 7-5-1, Kusunoki-Cho, Chuo-Ku, Kobe, 650-0017 Japan; 2 Department of Orthopaedic Surgery, Konan Medical Center, 1-5-16, Kamokogahara, Higashi Nada-Ku, Kobe, 658-0064 Japan

**Keywords:** Ankle osteoarthritis, Total ankle arthroplasty, Osteolysis, Component subsidence

## Abstract

**Purpose:**

This study aimed to investigate the relationship between periprosthetic osteolysis around the talar component and the amount of talar component subsidence after total ankle arthroplasty (TAA).

**Methods:**

This study included forty patients who underwent TAA with a mean follow-up of 67.5 ± 17.0 months. The patients were divided into two groups based on the amount of osteolysis around the talar component, as measured by computed tomography at the latest clinic visit: none to 2 mm (N group, n = 20) and greater than or equal to 2 mm (O group, n = 20). The average amount of talar component subsidence, clinical outcomes, and complications were compared between the two groups. In the O group, the correlation between osteolysis and talar component subsidence was evaluated.

**Results:**

The average talar component subsidence was significantly different between the N (0.22 ± 0.94 mm) and O groups (2.12 ± 2.28 mm). Five out of 20 ankles in the O group required revision surgery owing to talar component subsidence. The Japanese Society for Surgery of the Foot scores in the N and O groups were significantly different: 93.5 ± 7.7 and 85.3 ± 15.4, respectively. In the O group, we found that osteolysis tended to develop on the lateral side, and the amount of osteolysis was positively correlated with the talar component subsidence (*r* = 0.59, *P* = .007).

**Conclusion:**

In the O group, a positive correlation between osteolysis and talar component subsidence was found, and five patients required revision surgery.

**Supplementary Information:**

The online version contains supplementary material available at 10.1007/s00590-023-03519-9.

## Introduction

Total ankle arthroplasty(TAA) was first introduced in the 1970s as an alternative to ankle fusion (AF) to treat end-stage ankle arthritis [1]. AF was the preferred procedure for decades, as initial trials of TAA had high failure rates in the first- and second-generation models [2]. In the last decade, however, the number of TAA procedures has increased with implant design and survivorship improvements, and the complication rates have decreased compared with AF [3, 4]. One of the reasons for TAA failure is component subsidence, especially on the talar side. Multiple factors induced component subsidence as follows, aseptic loosening, malalignment of the components, disruption of the extraosseous or intra-osseous blood supply of the talus, avascular necrosis of the talus, and nonanatomic component design [5]. The osteolysis developing around the component was considered the principal cause of aseptic loosening. Previous studies reported that the rate of periprosthetic osteolysis ranged from 35 to 37% at the 3- to 4-year follow-up [6–8]. However, which osteolytic lesion could induce component subsidence is not clear. Early detection of such lesions may provide a clinical opportunity for limited revision surgery in ankles with impending prosthesis failure [6, 7]. Therefore, this study aimed to investigate the relationship between the amount of osteolysis in the talus and the amount of talar component subsidence. We hypothesized that a more significant osteolytic lesion would induce talar component subsidence and occasionally result in TAA revision.

## Methods

### Study design

This study retrospectively reviewed patients who underwent TAA between May 2012 and March 2018 at our hospital. All patients received a detailed explanation and consented to participate. The ethical committee of the Kobe University Graduate School of Medicine approved this study. The TAA was indicated for patients in a sedentary lifestyle, with symptomatic end-stage ankle osteoarthritis (OA) or rheumatoid arthritis (RA). The TNK ankle (Kyocera Medical, Kyoto, Japan), consisting of two aluminium ceramic components and a polyethylene surface, was used for all cases [9]. Forty-one patients underwent TAA during this period. Among these 41 patients, this study included the 40 patients who were followed up for more than 6 months postoperatively. One patient was excluded owing to death within 6 months. One experienced surgeon (N.K.) performed all procedures (Supplemental Fig. 1). Three weeks after surgery, the patients were discharged from the hospital and followed up every three months in the first year after surgery and every six months thereafter.

### Clinical outcomes

The Japanese Society for Surgery of the Foot scale (JSSF scale) was used for clinical outcome evaluation [10]. Higher points indicated better outcomes. Dorsiflexion and plantarflexion were assessed using a goniometer at every clinic visit after surgery. Complications during the follow-up period were recorded as well.

### Radiographic measurements

**Fig. 1:**
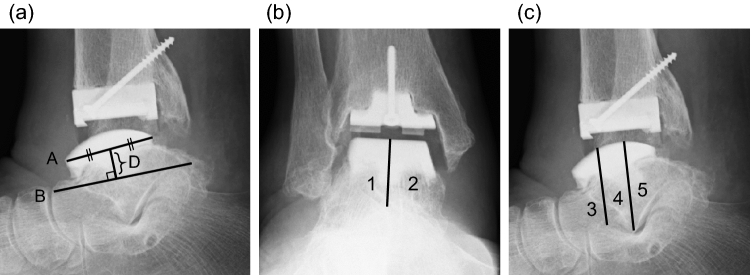
**a** Measurement of talar component subsidence using a sagittal plane radiograph. Line A was matched up with the talar component axis. Line B connected the dorsal aspect of the talar head and the tip of the posterior talar process. Line D was drawn perpendicular to Line B such that it meets Line A at its centre. The difference in the lengths of line D immediately after surgery and at the final follow-up was defined as talar component subsidence. **b** The zoning protocol for detecting locations of osteolysis. On the coronal plane radiograph, the talus was divided along the centre of the talar component, and the lateral and medial parts were assigned zones 1 and 2, respectively. **c** On the sagittal plane radiograph, the talar component was split into three parts vertically and the anterior, middle, and posterior regions were assigned zones 3, 4, and 5, respectively

Plain radiographs and computed tomography (CT) images were obtained at the latest clinic visit. The talar component subsidence was measured on a sagittal plane radiograph, as shown in Fig. 1.

CT detected each osteolytic lesion in the talus, and lesions larger than 2 mm were defined as positive, following the threshold reported by Rodriguez et al. [6]. In this study, 20 cases that had either no lesions or osteolytic lesions smaller than 2 mm were assigned to the N group, and the other 20 cases that had osteolytic lesions equal to or larger than 2 mm were assigned to the O group. The location of each osteolytic lesion was assigned to each zone based on the modified zoning protocol reported by Besse et al. (Fig. 1) [11]. In case the osteolytic lesion was extensive, and beyond the border, that lesion was assigned to the zone where a more significant part of the lesion existed. The correlations between the size of osteolysis measured by CT scan and the distance of subsidence of talar component were statistically evaluated.

### Statistical analysis

The results are presented as the mean ± standard deviation (SD). Statistical analysis was performed using GraphPad Prism (version 9.0; GraphPad Software, La Jolla, CA, USA). The differences between the parametric numbers of the N and O groups were analysed using Student's *t*-test. Proportional data were analysed using the chi-square test or Fisher’s exact test. Significance was set at *P* < 0.01. The correlations between the size of osteolysis and the talar component subsidence were evaluated using Pearson correlation tests.

## Results

In this study, the N group consisted of four males and 16 females, and the O group consisted of five males and 15 females (*P* = 0.705). The average body mass index (BMI) was 23.8 ± 2.7 kg/m^2^ in the N group and 22.8 ± 2.2 kg/m^2^ in the O group (*P* = 0.4315). The mean age at surgery of each group was 74.1 ± 4.7 years old in the N group and 69.8 ± 4.8 years old in the O group (*P* = 0.9763). The mean follow-up period of each group was 64.0 ± 17.6 months in the N group and 70.9 ± 15.1 months in the O group (*P* = 0.0812). The average amount of talar component subsidence of the N and O groups at the final follow-up were 0.55 ± 0.57 (range, 0–1.9) mm and 2.61 ± 1.95 (range, 0.7–8.5) mm, respectively, with a significant difference (*P* < 0.0001, Table 1).

**Table 1:** Patient characteristics

	N Group (*n* = 20)	O Group (*n* = 20)	*P*
Sex (male / female)	4 / 16	5 / 15	0.705
BMI (kg/m^2^)	23.8 ± 2.7	22.8 ± 2.2	0.4315
Age at surgery (months)	74.1 ± 4.7	69.8 ± 4.8	0.9763
Follow up (months)	64.0 ± 17.6	70.9 ± 15.1	0.0812
Subsidence (mm)	0.55 ± 0.57	2.61 ± 1.95	< 0.0001

Sex (*P* = .705), BMI (*P* = .4315), age at surgery (*P* = .9763), and follow up period (*P* = .0812) do not show significant difference between N group an O group. Talar Component subsidence is significantly large in O group (*P* < .0001)

The Takakura-Tanaka [12] classification was adopted to evaluate the stage of OA in each ankle. Among the 20 patients in the N group, two had RA and the rest had OA: two with stage 3a, 10 with stage 3b, and six with stage 4 OA. The 20 patients in the O group included: two of RA; two with stage 3a, 11 with stage 3b, and five with stage 4 OA. The distribution of patients in the two groups did not show a significant difference (*P* = 0.986, Table 2).

**Table 2:** Etiology and classification of OA

		N Group (*n* = 20)	O Group (*n* = 20)	*P*
Takakura/Tanaka OA stage classification	3a	2	2	.9868
	3b	10	11 (4)	
	4	6	5	
	RA	2	2 (1)	

The numbers in the enclosing indicate the cases that required revision surgery. The distribution between the two groups does not show a significant difference (*P* = .9868)

**Fig. 2:**
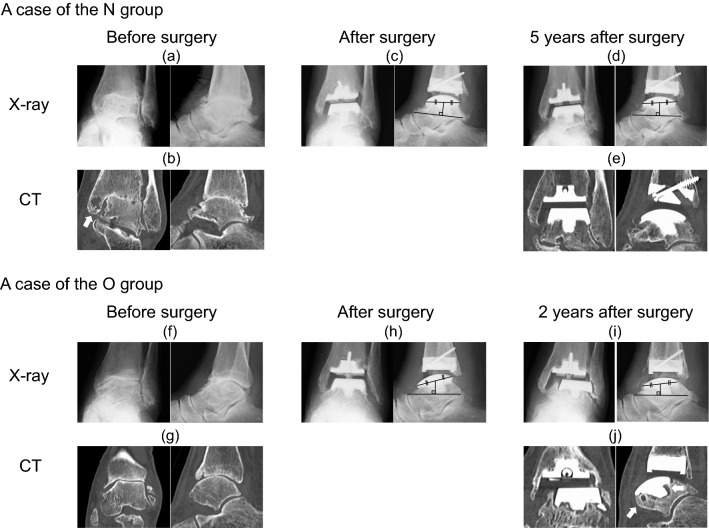
**a** A case of the N group. A-P and lateral X-ray images of osteoarthritis patient which were classified as Takakura/Tanaka classification stage 4. **b** Coronal and sagittal CT images revealed that joint space was almost disappeared and existence of bone cyst in medial malleolar(white arrow). **c** A-P and lateral X-ray images were taken immediately after TAA. Black lines were drawn to evaluate talar component subsidence, as described in Fig. 1. **d** A-P and lateral X-ray images were taken five years after surgery. Talar component subsided only 0.4 mm and the patient showed high JSSF score. **e** CT images revealed a few small osteolysis lesions. **f** A case of the O group who required revision surgery due to talar component subsidence. A-P and lateral X-ray images were classified as Takakura/Tanaka classification stage 3b. **g** Coronal and sagittal CT images revealed that joint space was narrowing and there was no large bone cyst in talus before surgery. **h** A-P and lateral X-ray images were taken immediately after TAA. Black lines were drawn to evaluate talar component subsidence. **i** A-P and lateral X-ray images were taken two years after surgery. Osteolysis development was not evident on X-ray, but the talar component subsided into the talus 2.3 mm deep. **j** CT images revealed some sizable periprosthetic osteolysis lesions (≥ 2 mm) developed in the talus. The patient received revision surgery due to an unstable and painful ankle

In terms of complications, delayed wound healing was observed in four ankles in the N group and two ankles in the O group. Fractures related to the surgical procedure occurred in five ankles: in the N group, one talar posterior process fracture and two medial malleolar fractures, and in the O group one lateral malleolar fracture and one medial malleolar fracture. In the O group, five female patients required revision surgery during the follow-up period owing to talar component subsidence, while the N group had no revision cases. The radiographic images of a patient in the N group and a patient in the O group who received revision surgery due to talar component subsidence induced by large osteolysis were displayed in Fig. 2. Five of revision cases consisted of one RA ankle, and four 3b OA ankles (Table 2).

**Fig. 3:**
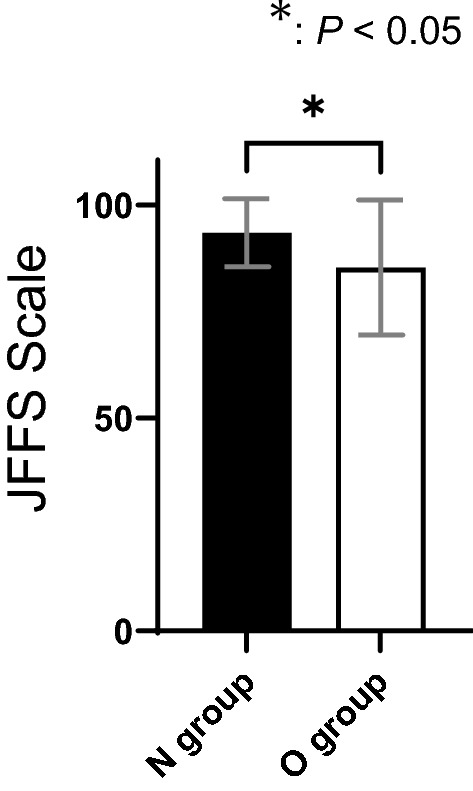
Comparison of clinical outcomes. The JFFS scale score. The JFFS scale score showed a significant difference

**Fig. 4:**
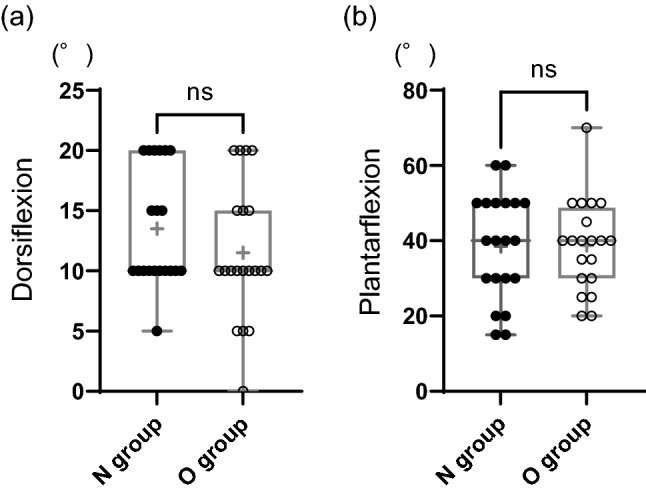
Measurement of **a** dorsiflexion angle and **b** plantarflexion angle. The ROM did not show significant difference in both dorsiflexion and plantarflexion

The average JSSF scale scores at the latest clinic visit for the N and O groups were 93.5 ± 7.7 and 85.3 ± 15.4, respectively, and there was a significant difference between the groups (*P* = 0.0042, Fig. 3). The average dorsiflexion angles of the N and O groups were 13.5 ± 4.8 degrees and 11.5 ± 5.5 degrees, respectively. The average plantarflexion angles of the N and O groups were 38.5 ± 13.8 degrees and 37.1 ± 9.6 degrees, respectively (Fig. 4). With the numbers available, No significant difference was detected in the ROM between the N and O groups with the numbers available.

In the O group, CT revealed the location of each osteolytic lesion. All 20 of the ankles in the O group had 22 osteolytic lesions equal to 2 mm or larger. Fourteen osteolysis lesions developed in zone 1 (lateral region), and eight lesions developed in zone 2 (medial region) on the coronal plane. Seven lesions were in zone 3 (anterior part), four lesions in zone 4 (middle part), and nine lesions in zone 5 (posterior part) on the sagittal plane (Table 3).

**Table 3:** The numbers of osteolysis in each location

Zone	2–5 mm	5–10 mm	Numbers
1: Lateral	7	7	14
2: Medial	6	2	8
3: Anterior	4	3	7
4: Middle	4	2	6
5: Posterior	5	4	9

Large osteolysis (5–10 mm) tend to develop in lateral side of talus

**Fig. 5:**
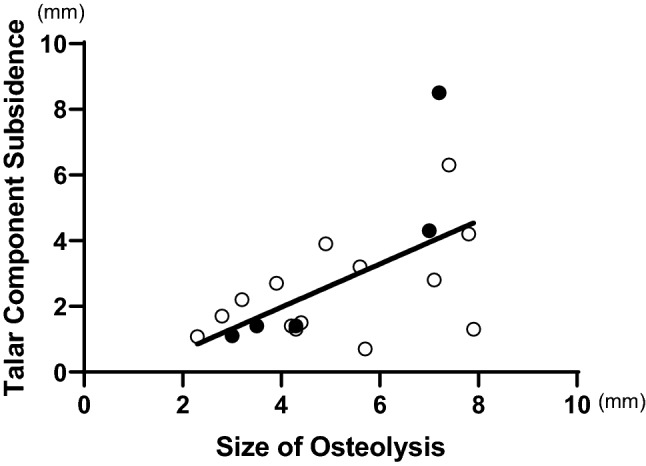
The correlation between the amount of osteolysis and talar component subsidence. A Pearson correlation test showed a moderate, positive, linear correlation that was significant between the amount of osteolysis and component subsidence (*r* = 0.59, *P* = .007). The black dots on the scattergram indicate revision cases in the O group

The relationship between the amount of osteolysis and subsidence showed a moderate, positive, linear correlation that was significant (*r* = 0.59, *P* = 0.007). The black dots on the scattergram represent revision cases in the O group (Fig. 5).

## Discussion

This study indicated that the talar component subsidence correlates with the amount of osteolysis. Notably, no cases showed more than 2 mm of subsidence in patients with small or no periprosthetic osteolysis (< 2 mm; N group), while the average subsidence was 2.61 mm in patients with sizeable periprosthetic osteolysis (≥ 2 mm; O group). This finding suggests a strong relationship between osteolysis and subsidence.

In our patients using the TNK ankle, five ankles in the O group required revision owing to talar component subsidence in an average 67.5 months follow up. The implant survival rate in our department was 88%, which was comparable to the reported mid-term results using Salto Talaris (Integra LifeScience, Plainsboro, NJ, USA), STAR (Stryker, Kalamazoo, MI, USA), and Agility (DePuy Orthopaedics, Warsaw, IN, USA) [13–15]. As previous papers reported higher revision rates in females after TAA, all five patients were female in this study [16]. The five revision cases consisted of a RA ankle and four of stage 3b OA ankle. Stage 3b OA ankles were characterised by partial subchondral bone contact with the inclined tibia-talar joint. Correcting severe coronal alignment deformity (> 20°) to neutral alignment through TAA was found to be associated with higher failure rates [2, 12, 17]. Coronal alignment deformity combined with large osteolysis may increase the risk of TAA failure, as observed in our cases.

The pathology of osteolysis development has been reported as following; polyethylene liner wear, high contact forces, implant incongruency, and micromotion of the implant [18]. Related to the contact force, poor bone quality, such as the talus low bone mineral density (BMD), could be another risk factor for osteolysis development and component subsidence. Lee et al. reported the relationship between BMD and osteolysis development after TAA by comparing the preoperative bone density of talus using Hounsfield units value in patients with and without periprosthetic osteolysis following TAA. The results showed no significant difference between patients with periprosthetic osteolysis and non-osteolysis, and the authors concluded that low bone density around the ankle joint might not be associated with development of osteolysis [19]. Furthermore, another paper reported that the bone density of the talus in patients with ankle OA undergoing TAA and those without OA did not  significantly differ [20]. The contribution of bone quality of talus on osteolysis development was still unclear.

Generally, osteolysis tends to enlarge after TAA with time, and the average follow-up period of the O and N groups was 70.9 months and 64 months, respectively, which were not enough long. We anticipated that osteolysis might develop even in the N group at a later period.

The average age of the N and O groups at surgery was 71.4 and 69.8 years, respectively, and there was no significant difference. Lee et al. compared clinical outcomes between patients younger than 55 and older who underwent TAA.They concluded that the complication rates, including component subsidence and revision, did not significantly differ between the two age groups [21]. The impact of age at the surgery on implant survivorship was not clear.

In this study, CT scans revealed osteolysis in the talus after TAA tended to develop on the lateral side. Increasing mechanical stress on the lateral side due to varus malalignment correction by TAA procedure has been reported to cause osteolysis [22]. Najefi et al. [23] reported that 78% of patients had a bone cyst outside the resection area prior to undergoing surgery in their series of 120 cases. Some osteolytic lesions in this study might have existed before surgery and remained after bone resection. A large subchondral bone cyst should be detected before surgery to reduce the risk of talar component subsidence.

TNK, the implant used in this study, is a fixed-bearing implant, and the selection of fixed-or mobile-bearing affects the development of osteolysis after TAA. Recently, Nunley et al. [24] reported comparing mobile-bearing and fixed-bearing TAA in a randomized control trial. According to their results, talar cyst formation and component subsidence occurred at more than ten times higher rates in mobile-bearing TAA.

TAA procedure showed excellent clinical outcomes in pain reduction and patient satisfaction [2, 25]. In this study, the ROM after TAA showed no significant difference between the two groups; however, the JFFS score was significantly lower in the O group owing to the pain sub-score of five patients who required revision. Most osteolytic lesions developed quiescently and were difficult to detect precisely with only plain radiographs [8]. Surgeons need to remember that subsidence progress to impending implant failure even though the patients are asymptomatic; hence, careful follow-up is required [5, 7, 26].

This study had several limitations. First, as mentioned above, the average follow-up period was not long enough. We will continue to follow up with patients to clarify the actual contribution of duration after TAA to the development of osteolysis. Second, this was a retrospective study; and in the future, we will need to conduct a prospective study to reduce several biases. Third, this study did not assess the bone quality of the talus before surgery, such as BMD. Although a study concluded that low bone density around the ankle joint might not be associated with the development of osteolysis; our cohort could show different results due to the difference in patient demographic background and implant design [19]. Lastly, this study did not evaluate tibial component alignment and surrounding osteolysis. Earlier studies reported that malalignment between the talar and tibial components induced incongruency and high focal pressure resulting in polyethylene liner wear. Among five revision cases in this study, two cases received both tibial and talar components replacement. Therefore, the tibial component should be analyzed, as well as the talar component, in future studies to reduce the failure of the TAA prosthesis.

## Conclusions

The number of patients who undergo TAA for end-stage ankle osteoarthritis has increased in the past decade. After surgery, osteolysis frequently developed around the implant without any symptoms. This osteolysis has been reported to cause implant subsidence, resulting in implant failure. This study showed a correlation between the amount of osteolysis and talar component subsidence. These results suggest that careful follow-up using images may enable surgeons to diagnose early and provide a clinical opportunity for limited revision surgery.

## Supplementary Information

Below is the link to the electronic supplementary material.

**Supplementary Fig.1.** The images showed a total ankle arthroplasty procedure. **a** The operation was performed using an anterior approach to the ankle. **b** Osteotomy of the tibial plafond and medial malleolus was performed perpendicularly to the anatomical axis of the tibia using a cutting guide. **c** Secondly, an osteotomy of the trochlea of the talus is performed in a direction parallel to the plantar surface. **d** The peg hole for the talar component fin is created using the chisel. **e** An osteotomy of the convex portion of the tibial component on the proximal side is performed. **f** Comfirming if osteotomy was precisely performed. **g** After implanting the talar and tibial component, an AO mini screw was inserted from a screw hole on the front face of the tibial component towards the direction of the posterior cortex for initial fixation. **h** After the movements of both components have been checked and confirmed, the retinaculum and the skin were sutured carefully (PPTX 12887 kb)
