# Supplementary figures and images for: Talar component subsidence correlates with periprosthetic osteolysis after total ankle arthroplasty
Source: Eur J Orthop Surg Traumatol. 2023 Mar 17;33(7):2987–93. doi: 10.1007/s00590-023-03519-9 (PMC10504111; doi:10.1007/s00590-023-03519-9)

## Slide 1
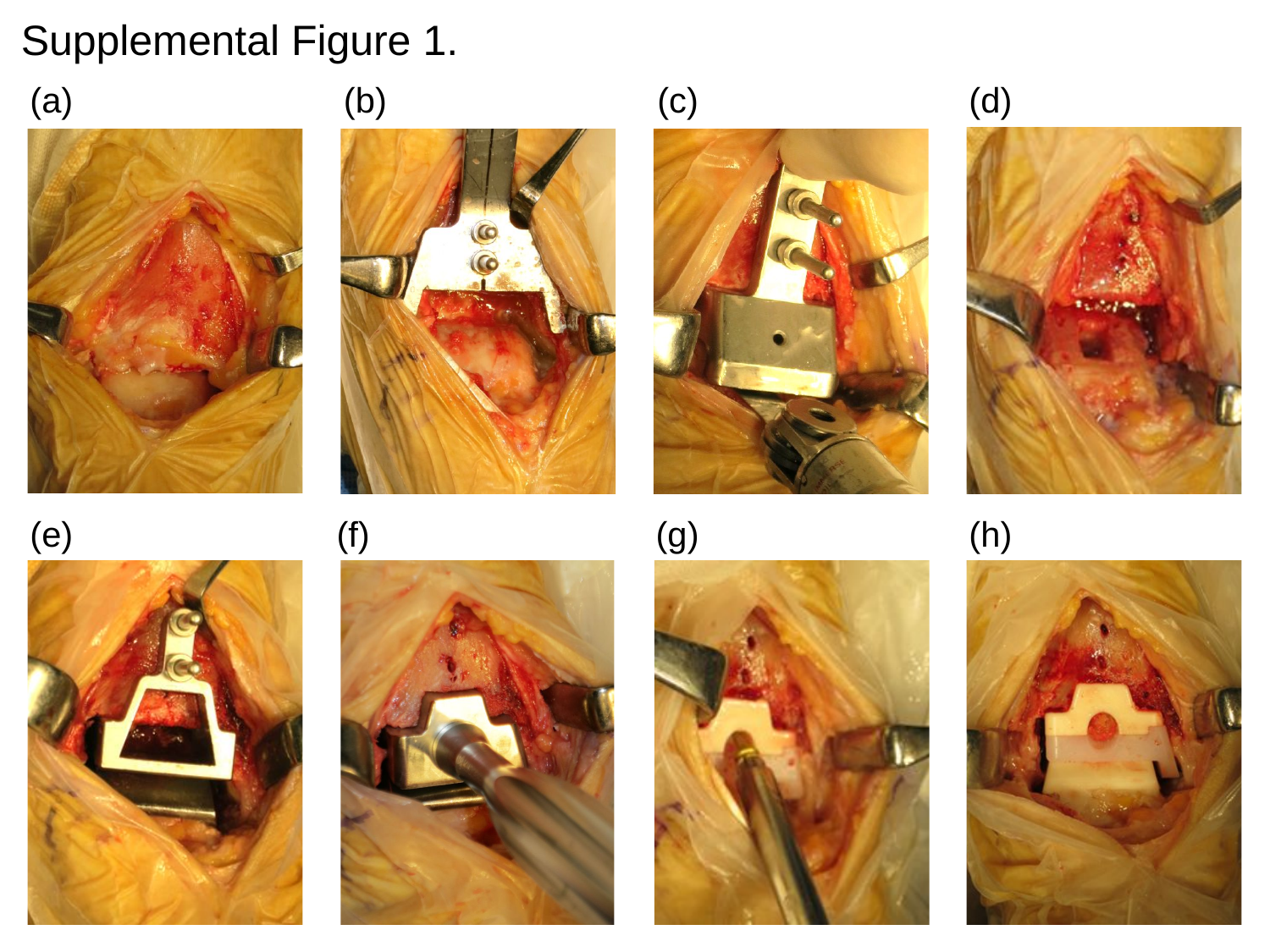

Supplemental Figure 1.
(a)
(b)
(c)
(d)
(e)
(f)
(g)
(h)

Supplement: Supplementary file 1 — Supplementary Fig.1. The images showed a total ankle arthroplasty procedure. a The operation was performed using an anterior approach to the ankle. b Osteotomy of the tibial plafond and medial malleolus was performed perpendicularly to the anatomical axis of the tibia using a cutting guide. c Secondly, an osteotomy of the trochlea of the talus is performed in a direction parallel to the plantar surface. d The peg hole for the talar component fin is created using the chisel. e An osteotomy of the convex portion of the tibial component on the proximal side is performed. f Comfirming if osteotomy was precisely performed. g After implanting the talar and tibial component, an AO mini screw was inserted from a screw hole on the front face of the tibial component towards the direction of the posterior cortex for initial fixation. h After the movements of both components have been checked and confirmed, the retinaculum and the skin were sutured carefully (PPTX 12887 kb) [file 590_2023_3519_MOESM1_ESM.pptx]
